# Effects of hyperthermia on the effective concentration of rocuronium and sugammadex-mediated reversal in isolated phrenic nerve hemidiaphragm preparations of rats

**DOI:** 10.1186/s12871-020-01114-7

**Published:** 2020-08-07

**Authors:** Jin Sun Kim, Young Mu Kim, Ha Jung Kim, Jae Moon Choi, Yong Beom Kim, Jae Seok Song, Hong Seuk Yang

**Affiliations:** 1 Department of Anesthesiology and Pain Medicine, Gangneung Asan Hospital, College of Medicine, University of Ulsan, Gangwon, Republic of Korea; 2 Department of Anesthesiology and Pain Medicine, Asan Medical Center, College of Medicine, University of Ulsan, Seoul, Republic of Korea; 3 Deaprtment of Anesthesiology and Pain Medicine, Gil Medical Center, College of Medicine, Gacheon University, Incheon, Republic of Korea; 4 Department Preventive medicine & Public Health Catholic Kwandong University, Gangneung, Republic of Korea; 5 Department of Anesthesiology and Pain Medicine, Sun General Hospital, Daejeon, Republic of Korea

**Keywords:** Antagonist, Sugammadex, Neuromuscular blockade, Rocuronium, Hyperthermia

## Abstract

**Background:**

Hyperthermia is relatively rare during general anesthesia; however, a few studies have been conducted on hyperthermia and the neuromuscular blockade (NMB) induced by rocuronium, and the reversal of NMB by sugammadex. We investigated the effect of hyperthermia status on the NMB induced by rocuronium, and its reversal by sugammadex, in isolated phrenic nerve hemidiaphragm (PNHD) preparations of the rat.

**Methods:**

Thirty-three male Sprague-Dawley rat PNHD preparations were randomly assigned to three groups at different temperatures (36 °C, 38 °C, and 40 °C; each group, *n* = 11, in Krebs solution). The train-of-four (TOF) and twitch height responses were checked mechanomyographically. The PNHD were treated with progressively increasing doses of rocuronium and three effective concentrations (ECs), EC50, EC90, and EC95, of rocuronium were analyzed in each group via nonlinear regression analysis. Then, sugammadex was administered in doses equimolar to rocuronium. Thereafter, the T1 height (%), TOFR (%) and the duration index were measured.

**Results:**

The EC of rocuronium (EC50, EC90, and EC95) decreased significantly in accordance with increasing temperature. The groups at 36 °C and 40 °C showed clear differences in all areas (all *P* < 0.001). Moreover, the T1 height (%) and the duration index upon sugammadex administration showed faster recovery results in the36 °C than the 38 °C and 40 °C groups.

**Conclusion:**

A rise of temperature from 38 °C to 40 °C in rat PNHD preparations proportionally enhanced the NMB induced by rocuronium. In addition, equimolar doses of sugammadex to the administered rocuronium showed a slower recovery time as the temperature rises.

## Background

In general, body temperature decreases during anesthesia due to the administration of anesthetic agents and the environment of the operating room [1, 2]. Cases of hyperthermia exceeding the range of normal body temperature during general anesthesia are relatively infrequent [3]. Changes in body temperature during an operation result in changes in the pharmacodynamics of drugs such as neuromuscular blocking agents or agents that reverse neuromuscular blockade (NMB) such as sugammadex. Therefore, the use of these drugs requires an abundance of caution during emergencies requiring anesthesia.

However, clinical studies investigating hyperthermia are still unavailable and large-scale investigations are not feasible due to ethical constraints. Therefore, we designed specific environmental conditions at hyperthermic temperatures of 38 °C and 40 °C under in vitro conditions to demonstrate the effects of rocuronium and sugammadex using the phrenic nerve hemidiaphragm (PNHD) of rats.

## Methods

### Ethical statement

After ethical approval was granted by the Institutional Animal Care and Use Committee of Asan Medical Centre, Seoul, South Korea (approval number 2016–13–067 on 3 March 2016; Chairperson Professor Jong Yeun Park), 33 male Sprague–Dawley rats weighing 238–256 g were provided by the Animal Care and Use of Asan Medical Centre, College of Medicine, University of Ulsan (Seoul, Korea).

A random number was generated using the Microsoft Office Excel 2013 program.

All mice were bred in the laboratory animal breeding room at the Laboratory Animal Research Center, Asan Institute for Lifesciences. The animals were housed in an individually ventilated cage system (Tecniplast, USA) under specific pathogen-free conditions with a 12-h light/12-h dark cycle (from 8 am to 8 pm) at the following conditions: temperature 22 ± 1 °C, humidity 50 ± 10%, laboratory rodent chow, and reverse osmotic water.

### Experimental procedures

Thirty-three rats were randomly assigned to three groups at different temperatures (36 °C, 38 °C, and 40 °C; each group, *n* = 11). Sorting was accomplished using a random number generator in Microsoft Excel 2013 (Microsoft, Redmond, WA, USA). The rats were anesthetized via intraperitoneal injection of 30 mg/kg zoletil 50 (Virbac, Carros, France). Each anesthetized rat was bled and the diaphragm was extracted after checking for cardiac arrest. The thoracic cages were separated en bloc, and the left phrenic nerve-hemidiaphragm (PNHD) was prepared. The phrenic nerve-hemidiaphragm was fixed vertically in a 75 mL organ bath containing Krebs buffer (pH 7.4;NaCl 118 mM, KCl 5.0 mM, CaCl2 2.5 mM, NaHCO3 30 mM, KH2PO4 1.0 mM, MgSO4 1.0 mM, and glucose 11.4 mM). The bath was preserved at 36 °C, 38 °C, or 40 °C via external warm water circulation before fixing the PNHD followed by continuous inflow of 95% O2 and 5% CO2 into the Krebs solution. A rib of each specimen was connected to bipolar platinum electrodes. The tendinous portion of the hemidiaphragm was connected to a force-displacement transducer (Grass FT03, Grass Instrument Co., Quincy, Massachusetts, USA) to measure isometric contraction at a resting tension of 2 g using a nerve stimulator (S88, Grass Instrument Co., Quincy, Massachusetts, USA). A stimulus isolation unit (SIU5, Grass Instrument Co. Quincy, Massachusetts, USA) with train of four (TOF) pulses of 2 Hz was also used to provide a supramaximal stimulation of 0.2 ms pulse duration at intervals of 20s. The muscle contraction responses were registered and digitized with a Power Lab acquisition system and stored on a computer using data charting software (LabChart, ADInstruments).

In all groups, after more than 30 min of stabilization, the contraction response to initial TOF stimulation, the height of the first twitch, and TOF ratio were measured. At least 20 min was allowed to achieve a stable state before rocuronium was added to the organ bath at an initial loading dose of 200mcg. The T1 heights just before the addition of rocuronium served as the control values for each group. A booster dose of 150mcg was cumulatively added every 10 min until the first twitch response disappeared completely. The concentrations of rocuronium that generated depression of the first twitch to 5, 10, 25, 50, 75, 90, and 95% of control were defined as the effective concentrations (EC) of rocuronium, ranging from EC5 to EC95. All the experimental drugs were injected into the chamber using an air displacement micropipette, and 10 min after the T1 height was completely suppressed, equimolar doses of sugammadex were injected into the organ bath to evaluate the recovery of diaphragmic muscle contractions. The T1 height and the TOF ratio (TOFR) at every 5 min until 30 min recovery was observed after the administration of sugammadex and defined as the recovery period. The following parameters were measured: the time for the first twitch to 25% of the control (duration, 25%); the time to recovery of the first twitch from 25 to 75% of the control (duration, 25 to 75%); the recovery index (the time to recovery from 25 to 75% of the control value of T1 height); the time taken for T1 to attain 95% of the control T1 height (the 95% T1 time); the time taken for the TOFR to reach 0.9 (the TOFR 0.9 time); and the number of samples that failed to reach TOFR 0.9 in each group.

### Statistical plan

To determine the required sample size, the time from sugammadex administration to TOFR 0.9 in three groups (36 °C, 38 °C, and 40 °C in the organ bath, each *n* = 3) was measured in a pilot study. The time for the three groups was 18.8 ± (3.2), 25.2 ± (4.3), and 25.9 ± (3.0) min, respectively (36 °C, 38 °C, and 40 °C, respectively).

The effect size (f) calculated using Cohen’s formula was 0.6.R, indicating that 10 specimens in each group yielded α = 0.05, power = 0.8, and f = 0.6. We used 11 specimens in each group to compensate for dropout.

The results are expressed as mean ± SD. Normality of the continuous variables was assessed using the Shapiro-Wilk test. Differences in the doses of rocuronium administered, ECs, T1 height (%), TOFR (%), 95% T1 times, and TOFR 0.9 times among the groups were assessed using Kruskal-Wallis test with Bonferroni correction. The ECs of rocuronium were calculated via nonlinear regression analysis. All statistical analyses were conducted with the aid of SPSS® software (ver. 18.0, SPSS Inc., USA). *P* values < 0.05 were regarded as statistically significant.

## Results

**Fig. 1:**
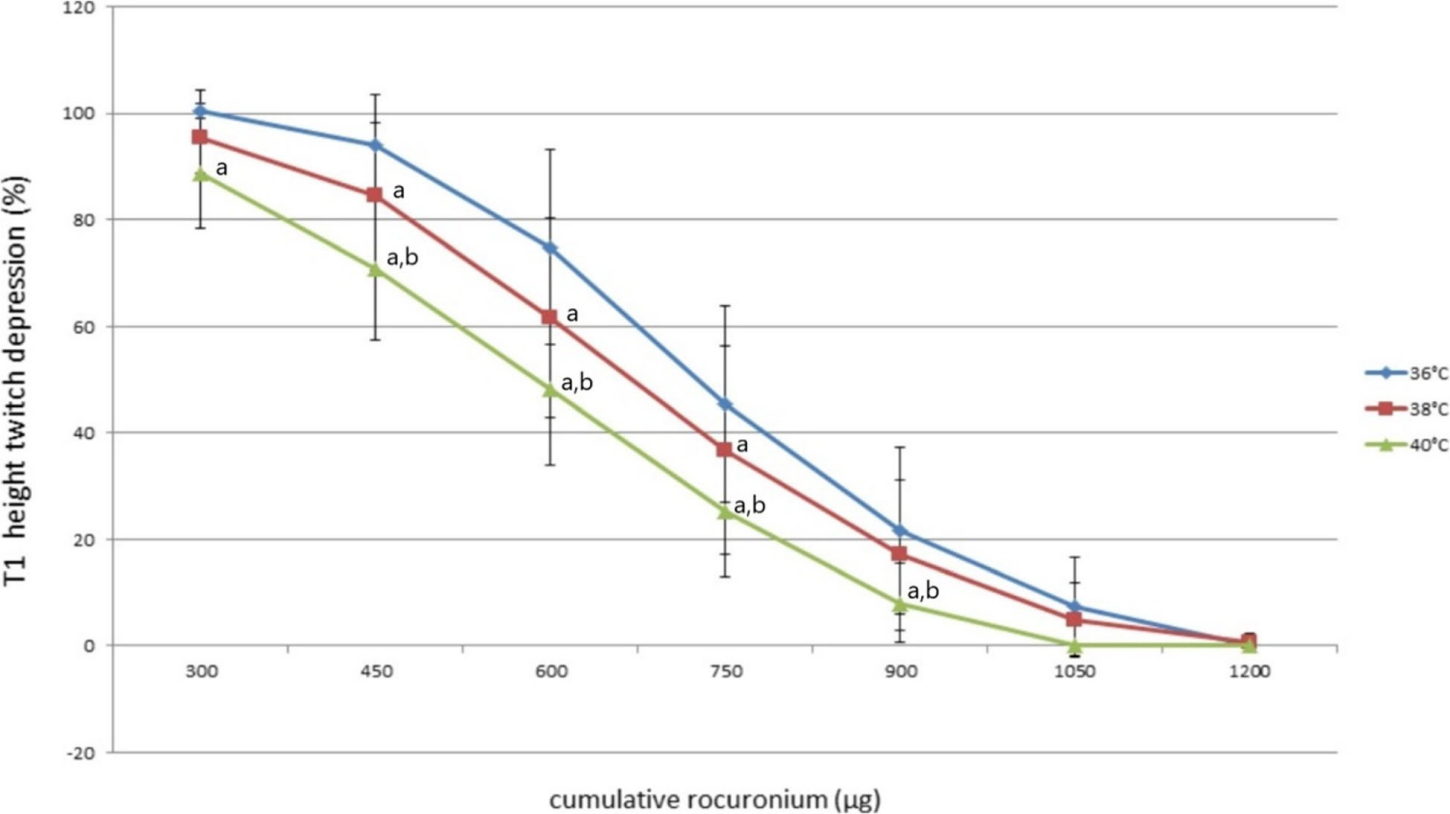
Cumulative concentration-response curves of rocuronium in the three groups. Data are presented as mean ± SD. T1 height (%) exhibited a significant decline in the 450 μg- 750 μgphase of administration; especially the 36 °C and 40 °C groups showed clear differences in all areas (*P* = 0.019). **a***P* < 0.05 vs. 36 °C (Bonferroni corrected); **b** P < 0.05 vs. 38 °C (Bonferroni corrected)

The mean size and weight of the PNHD tissue preparations of the groups at 36 °C, 38 °C, and 40 °C were not different (Table 1). Comparison of the rocuronium concentration responses revealed that the T1 height (%) exhibited a significant decline as the temperature increased in the 36 °C, 38 °C, and 40 °C groups, especially the groups at 36 °C and 40 °C, which showed clear differences in all areas (*P* = 0.019) (Fig. 1). The cumulative concentration-response curves of rocuronium for the three groups shifted to the left as the temperature increased by 2 °C.

**Table 1:** The mean size and weight of the phrenic nerve-diaphragm tissue

Group	Rat body weight(g)	Hemi-diaphragm mass (mg)	Hemi-diaphragm length (mm)	Hemi-diaphragm width (mm)
36 °C(*n* = 11)	245.7 (65.3)	168.8 (49.3)	10.2 (1.3)	19.3 (2.7)
38 °C(n = 11)	256.8 (34.2)	163.9 (29.6)	9.6 (0.9)	20.1 (3.4)
40 °C(n = 11)	238 (18.9)	152 (25.3)	8.8 (1.1)	17.2 (2.1)

Data are expressed as mean ± SD

Differences existed in effective concentration (EC) between the three groups (all *p* < 0.0015) (Table 2). The EC50 of rocuronium was significantly reduced in the 40 °C group compared with the groups at 36 °C and 38 °C (*P* = 0.0005 and *P* = 0.0356, respectively). As the temperature increased, the EC10, EC25, EC50, EC75, EC90, and EC95 of rocuronium significantly decreased in the group at 40 °C compared with the group at36 °C (all *P* < 0.001). A significant difference was observed between the groups at 38 °C and 40 °C (*P* = 0.045), although it was smaller than the difference between the groups at 36 °C and 40 °C.

**Table 2:** Comparisons of effective concentration of rocuronium

	36 °C	38 °C	40 °C	*P*-value among all the groups	36 vs. 38	38 vs. 40	36 vs 40
EC5(μg/)	5.3 (1.3)^a^	4.2 (1.2)	3.1 (1.0)^c^	0.0014	0.048	0.066	0.0006
EC10	5.8 (1.2)	4.8 (1.2)	3.7 (1.0)^c^	0.0014	0.056	0.056	0.0006
EC25	6.5 (1.1)	5.6 (1.1)^b^	4.5 (0.9)^c^	0.0015	0.087	0.041	0.0006
EC50	7.2 (1.0)	6.4 (1.1)^b^	5.3 (0.8)^c^	0.0014	0.115	0.035	0.0005
EC75	7.9 (0.9)	7.2 (1.0)^b^	6.2 (0.8)^c^	0.0014	0.167	0.035	0.0004
EC90	8.6 (0.9)	8.0 (1.0)_b_	7.0 (0.7)^c^	0.0011	0.131	0.035	0.0003
EC95	9.1 (0.8)	8.5 (1.0)^b^	7.6 (0.6)^c^	0.0015	0.167	0.048	0.0003

Values presented as mean (SD). EC, effective concentration

^a^P value less than 36 °C vs. 38 °C

^b^P value less than 38 °C vs. 40 °C

^C^P value less than 36 °C vs. 40 °C

**Fig. 2:**
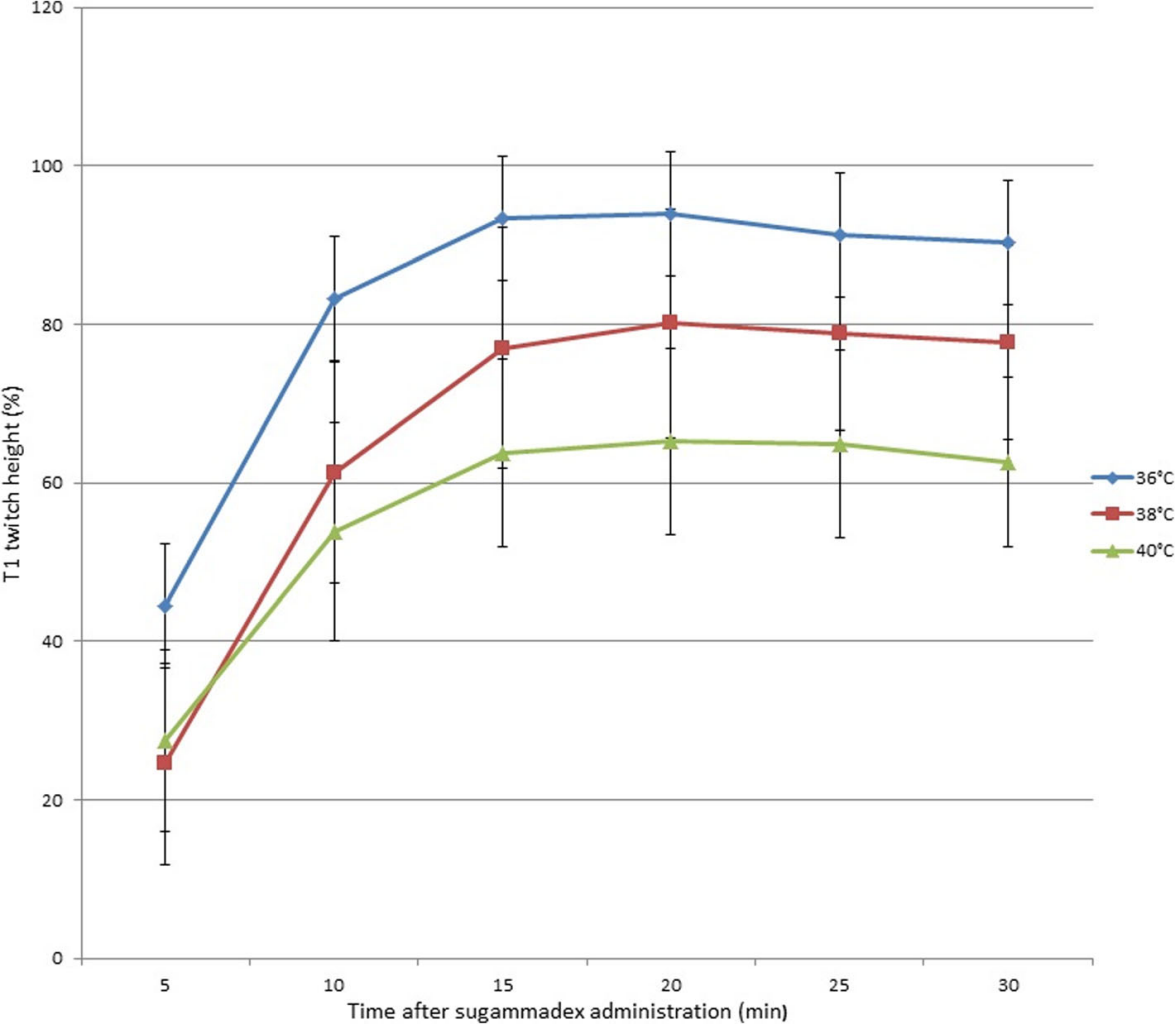
Changes in the T1 height (%) duration during the 30 min recovery period significantly differed between the groups. The group at 36 °C showed increased recovery than did the groups at 38 °C and 40 °C(*P* = 0.011, *P* < 0.001 respectively)

**Fig. 3:**
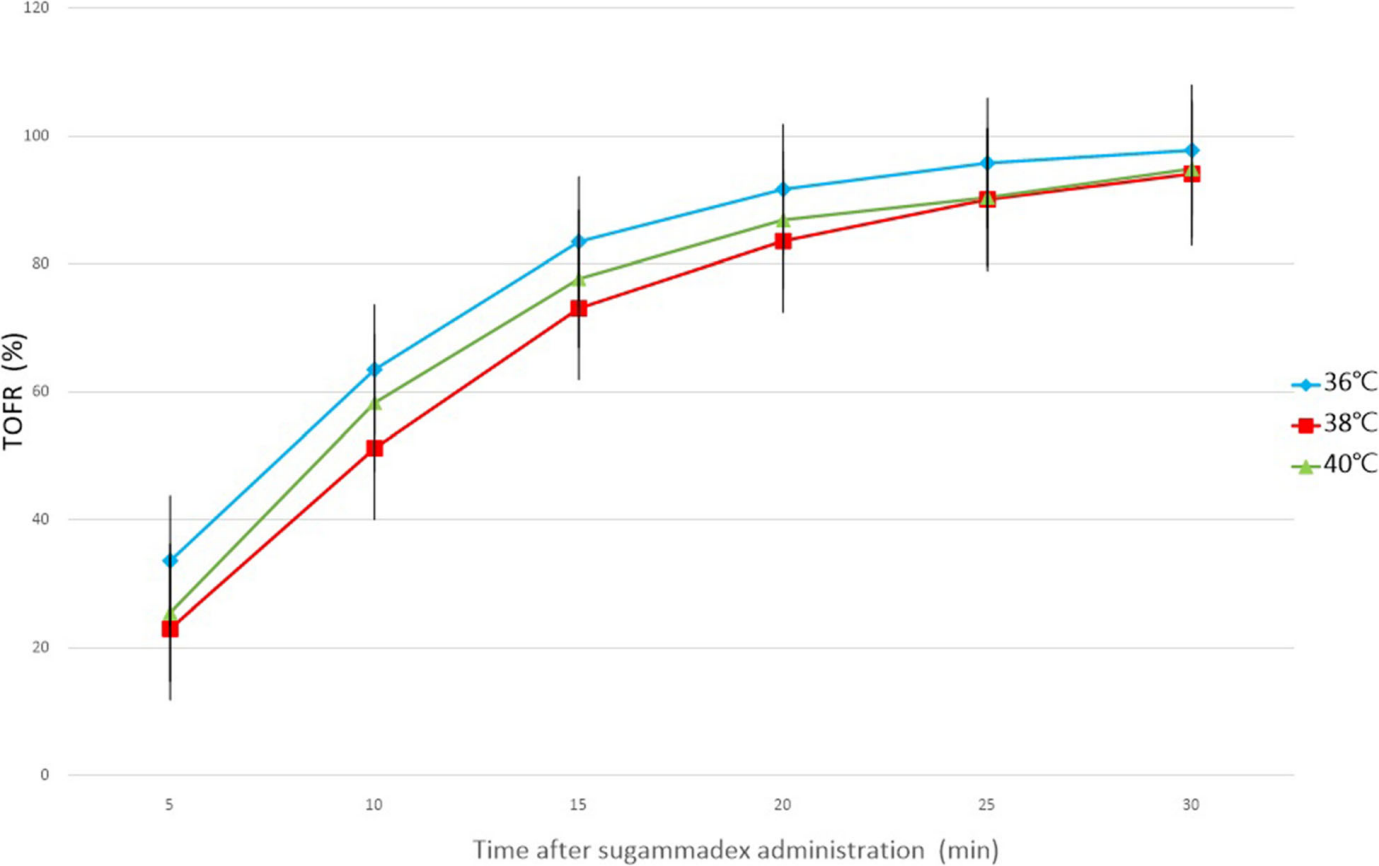
TOFR 0.9 of control exhibited no difference among the groups after administration of equimolar doses of sugammadex

Measurement of the T1 height (%)after the administration of sugammadex at doses equimola to the rocuronium revealed that the group at 36 °C showed significantly faster recovery than did the groups at 38 °C and 40 °C (*P* = 0.011, *P* < 0.001, respectively) (Fig. 2). The groups at 38 °C and 40 °C did not show substantial differences in the first 10 min; however, after that the T1 heights of the group at 40 °C were significantly shorter than those of the group at 38 °C (*p* = 0.01). Pair-wise comparisons showed no differences in the recovery of TOFR (%) among the groups (Fig. 3).

The duration index (25to 75%) was distinctly higher in the 36 °C group than in the groups at 38 °C and 40 °C (*P* = 0.0113 and *P* < 0.0001, respectively) (Table 3). The maximum T1 height during 30 min showed a significant shortening in the 40 °C group compared with the groups at 36 °C and 38 °C. All the samples reached TOFR 0.9 after sugammadex administration.

**Table 3:** Twitch height and train-of-four ratio after the addition of sugammadex

	36 °C	38 °C	40 °C
Maximum T1 height	16.1 (3.7)	16.5 (4.1)	13.1 (2.6)
Dur 25 (%) min	2.1 (0.8)	2.7 (0.8)	5.0 (1.7)
Dur 25 ~ 75 (%) min	3.7 (1.3)	4.7 (0.8)	5.1 (1.0)
TOFR (%) in 30 min	97.8 (3.6)	94.1 (6.0)	94.9 (5.8)
Maximal TOF ratio < 0.9(n)	0	0	0

Values are expressed as mean ± (SD) or number, TOF: train-of-four, T1 height of train-of-four, Dur 25%: time from reversal to recovery of T1 to 25%, Dur 25 to 75%: time to recovery of T1 from 25 to 75%

## Discussion

During neuromuscular blockade of PNHD using rocuronium, the T1 height (%) was significantly decreased as the temperature increased from 36 °C to 40 °C at same doses. The cumulative concentration-response curve of rocuronium in each group shifted to the left with every 2 °C rise in temperature. Also, the EC of rocuronium decreased following the rise in temperature. Addition of equimolar doses of sugammadex to rocuronium resulted in a faster recovery in the 36 °C group compared with the other groups. Also, the T1 heights (%) of the 38 °C and 40 °C groups failed to recover sufficiently for 30 min.

Several factors contribute to a perioperative rise in body temperature. The general causes include fever associated with infection, blood transfusion mismatch, drug toxicity, and allergic reactions excluding genetic diseases such as malignant hyperthermia [3]. Moreover, hyperthermia can easily occur in infants upon excessive heating when core temperature monitoring devices are not used [4].

Analysis of the effects of NMB agents on hyperthermia is complicated by two factors. The effects of higher body temperature on NMB need to be distinguished from those of NMB agents. Also, the distinct role of each of the pharmacokinetic and pharmacodynamic factors should be elucidated.

The tension associated with a muscular twitch involves two components: internal shortening and relaxation decay [5]. The reaction decreases in accordance with decreasing temperature [6]. Therefore, at a lower temperature, the interaction between actin and myosin filaments is delayed, which is related to a temperature-dependent removal of intracellular calcium [7, 8]. Increasing the muscle temperature to the level of core temperature resulted in a decrease in calcium sensitivity and increase in reactive oxygen species level [9, 10]. Changes in calcium sensitivity and reactive oxygen species can affect both pre- and post-synaptic sites [11, 12]. Thus, changes in twitch tension can arise depending on varying levels of temperature in cases unexposed to NMB agents. However, data on human neuromuscular junctions at temperatures above 36 °C are not sufficient yet.

Our findings only pertain to the effect of temperature on muscle tension and contraction. In vivo physiological changes due to hyperthermia during the perioperative period, such as altered cardiac output and changes in regional blood flow may induce changes in drug metabolism. Furthermore, changes in temperature can alter the pharmacokinetics and pharmacodynamics of NMB and reversal agents [13, 14]. Under hypothermia, the duration of NMB in humans is prolonged [15, 16]. Similarly, hyperthermia has been reported to shorten NMB duration, possibly due to increased hepatic uptake [17]. While in vitro methodologies have been shown to distinguish the effects on muscle physiology from pharmacokinetic and dynamic factors, the interplay between these factors and those identified in vitro is unknown in an intact organism.

We used an equimolar dose of sugammadex to determine the effect of hyperthermia on the reversal of NMB. Few studies have explored the pharmacodynamics of sugammadex in hyperthermia. However, in a previous study in which NMBs were induced by rocuroniumin in two groups of patients, one normothermic and one hypothermic, and the patients were treated with sugammadex, the group with slight hypothermia (34.5–35 °C) and the group at normal temperature (36.5–37 °C) experienced reversal from a state of deep NMB (PTC 1–2) following sugammadex (4.0 mg/kg) treatment, with a faster recovery of the normal group by 46 s compared with the hypothermia group [18]. The investigators reported that the difference was caused by decreased plasma clearance in the low-temperature group although their study lacked clinical significance. Based on this finding, we predicted that our results would show a similar recovery in the hyperthermia group compared with the normal temperature group when NMB was reversed by sugammadex. However, it was found that the recovery of the 36 °C group was significantly faster compared with the 38 °C and 40 °C groups. In addition, the recovery index was faster in the 36 °C group compared with the other groups. Moreover, theT1 heights (%) of the 38 °C and 40 °C groups did not recover adequately for 30 min. Nevertheless, the time taken to reach TOFR 0.9 was not influenced by temperature. Interestingly, similar to clinical recovery of NMB with a low dose of sugammadex, the T1 height (%) peaked earlier, and the TOFR 0.9 was observed later [19]. It can be assumed that free rocuronium, which is not removed by sugammadex, binds to postsynaptic acetylcholine receptors more strongly during the recovery period [20, 21]. It is possible that hyperthermia may have interfered with the combination of sugammadex and rocuronium.

It is speculated that the function of acetylcholine or cholinesterase is probably affected by hyperthermia. However, other studies using the PNHD of rats showed an increase in temperature up to 43 °C following muscle usage or environmental factors [22], and the threshold value of temperature that can be reversed without damage was 45.3 °C, which is consistent with the experimental conditions [23]. In addition, the acetylcholine association and dissociation rate constants were independent at a wide range of temperatures [24]. Therefore, the temperature range of 38 °C to 40 °C does not have a significant effect on the function of acetylcholine.

The study had the following limitations: First, it was an in vitro test that failed to analyze pharmacodynamic and pharmacokinetic effects simultaneously; second, it failed to monitor the histological and pathophysiological changes of muscles induced by changes in temperature.

## Conclusion

An increase in temperature from 38 °C to 40 °C in rat PNHD preparations proportionally enhanced the NMB induced by rocuronium. In addition, equimolar doses of sugammadex to the administered rocuronium showed a slower recovery time as the temperature increased.

## Data Availability

The datasets used and/or analyzed during the current study are available from the corresponding author upon reasonable request.

## Abbreviations

NMB: neuromuscular blockade
PNHD: phrenic nerve hemidiaphragm
TOF: train-of-four
EC: effective concentration
TOFR: train-of-four ratio
